# Susceptibility of Carrion Crows to Experimental Infection with Lineage 1 and 2 West Nile Viruses

**DOI:** 10.3201/eid2108.140714

**Published:** 2015-08

**Authors:** Stephanie M. Lim, Aaron C. Brault, Geert van Amerongen, Angela M. Bosco-Lauth, Hannah Romo, Varsha D. Sewbalaksing, Richard A. Bowen, Albert D.M.E. Osterhaus, Penelope Koraka, Byron E.E. Martina

**Affiliations:** Erasmus Medical Center, Rotterdam, the Netherlands (S.M. Lim, G. van Amerongen, V.D. Sewbalaksing, A.D.M.E. Osterhaus, P. Koraka, B.E.E. Martina);; Centers for Disease Control and Prevention, Fort Collins, Colorado, USA (A.C. Brault, A.M. Bosco-Lauth, H. Romo);; Colorado State University, Fort Collins (R.A. Bowen);; Artemis One Health Research Institute, Utrecht, the Netherlands (A.D.M.E. Osterhaus, P. Koraka, B.E.E. Martina)

**Keywords:** West Nile virus, WNV, carrion crow, Europe, corvid, susceptibility, virulence, viruses, experimental infection, surveillance, sentinel, Corvus corone

## Abstract

West Nile virus (WNV) outbreaks in North America have been characterized by substantial die-offs of American crows (*Corvus brachyrhynchos*). In contrast, a low incidence of bird deaths has been observed during WNV epidemic activity in Europe. To examine the susceptibility of the western European counterpart of American crows, we inoculated carrion crows (*Corvus corone*) with WNV strains isolated in Greece (Gr-10), Italy (FIN and Ita09), and Hungary (578/10) and with the highly virulent North American genotype strain (NY99). We also inoculated American crows with a selection of these strains to examine the strains’ virulence in a highly susceptible bird species. Infection with all strains, except WNV FIN, resulted in high rates of death and high-level viremia in both bird species and virus dissemination to several organs. These results suggest that carrion crows are highly susceptible to WNV and may potentially be useful as part of dead bird surveillance for early warning of WNV activity in Europe.

## Abstract 2

These birds are highly susceptible to strains circulating in Europe and, thus, may serve as surveillance sentinels.

West Nile virus (WNV), a flavivirus (family *Flaviviridae*) transmitted by mosquitoes, uses birds as its primary vertebrate reservoir host. WNV has an extensive geographic range that includes Europe, Africa, the Middle East, southern Asia, and Australia (*1*). In 1999, WNV emerged in North America, where it was first detected in New York, New York. The virus subsequently spread rapidly across the continent, becoming the leading cause of arboviral encephalitis in humans and horses (*2*), and it was associated with deaths among at least 326 bird species (*3*). High death rates are most frequently observed among passeriform birds, of which the family *Corvidae* comprises the most highly susceptible species to WNV (*4*). In particular, deaths among the American crow (*Corvus brachyrhynchos*) have been used to track the spread of the virus across many parts of North America (*5*–*8*).

Since 2008, WNV has been responsible for outbreaks throughout central and southeastern Europe, affecting countries such as Greece, Italy, Hungary, Romania, and Croatia and constituting a serious veterinary and public health problem. Fatalities have been reported among wild birds in Europe, such as eagles (*9*,*10*), sparrow hawks, goshawks, geese, and falcons (*11*–*13*). However, death rates among birds in Europe have been low, and no clustered death events have occurred, even when cases were associated with outbreaks of severe human and equine WNV infections (*14*–*17*). Several theories have been proposed to explain the low death rates among birds in Europe: limited or insufficient monitoring of deaths among wild birds in Europe; development of immunity among birds from infections acquired on wintering grounds (*18*); and circulation of WNV strains in Europe with reduced virulence for birds.

Experimental infection of American crows with the North American genotype of WNV (NY99) has shown that the strain has a highly pathogenic phenotype: viremia titers exceeded 9 log_10_ PFU/mL, and all infected birds died (*19*–*23*). However, the lack of WNV-associated bird deaths in Europe suggests that European birds might not be susceptible to WNV or that WNV strains from Europe are not virulent to birds. Thus, we evaluated the susceptibility of the European equivalent of the American crow, carrion crows (*Corvus corone*), which are ubiquitously present across Europe, by injecting them with selected strains of WNV circulating in Europe and with the prototypic NY99 strain. In addition, we inoculated American crows with a selection of these viruses to assess and compare the virulence of WNV strains from Europe in a bird species known to be highly susceptible to WNV. Susceptibility was assessed in terms of death, survival time, magnitude and duration of viremia, and spread of virus to different organs.

## Materials and Methods

### Source of Virus and Birds

Five different WNV strains were used in this study: lineage 1a strain NY99-4132 (NY-99) (*20*); lineage 2 strain Nea Santa-Greece-2010 (Greece-10; GenBank accession no. HQ537483.1) (*24*); lineage 1a strain Italy/2009/FIN (FIN; GenBank accession no. KF234080); lineage 1a strain Ita09 (GenBank accession no. GU011992.2) (*25*); and lineage 2 strain 578/10 (GenBank accession no. KC496015). Further details about these viruses are provided in Table 1.

**Table 1:** West Nile virus strains used for susceptibility studies in carrion and American crows

Strain	Source	Passage history*	Location	Genetic lineage	Crow species inoculated
NY99-4132	American crow (brain)	V2	United States	1a	Carrion, American
Nea Santa-Greece-2010	*Culex pipiens* mosquito	V1	Greece	2	Carrion
Italy/2009/FIN	Human with neuroinvasive disease	V2, C1	Italy	1a	Carrion, American
Ita09	Human with neuroinvasive disease	V1, C1	Italy	1a	Carrion, American
578/10	Horse (brain)	V2, C1	Hungary	2	Carrion

*Viruses were propagated in Vero (V) or C6/36 insect cells (C). Numbers following passage source represent the number of virus passages.

Carrion crows were captured by using walk-in traps in the municipality of Rotterdam, the Netherlands, and then transported to indoor housing at the animal holding facilities at the National Institute for Public Health and the Environment, Bilthoven, the Netherlands. After being inoculated with WNV, the crows were cared for in groups of 8 in isolators under negative pressure. Only seronegative birds were used in this study. Seronegativity was determined by using a neutralization assay (Technical Appendix).

American crows were captured by using cannon net traps in Bellvue, Colorado, USA; the National Wildlife Diseases Program, Animal and Plant Health Inspection Service, United States Department of Agriculture, assisted with the captures. The crows were banded and transported to Fort Collins, Colorado, where they were housed in 1-m^3^ cages (2 birds per cage) at the Colorado State University Animal Disease Laboratory.

### Experimental Infection and Sampling Protocol

Crows were subcutaneously inoculated in the thigh or breast region with 2,000 50% tissue culture infectious doses (TCID_50_) of virus per 0.1 mL of serum-free Dulbecco’s Modified Eagle Medium (DMEM) (Lonza Benelux BV, Breda, the Netherlands). Carrion crows (8 per virus) were injected with WNV strain NY99, Greece-10, FIN, Ita09, or 578/10. American crows were inoculated with NY99 (n = 6), FIN (n = 5), or Ita09 (n = 5). Approximately 0.1 mL of blood was collected from carrion crows at 2-day intervals, up to 8 days postinoculation (dpi), and 0.2 mL of blood was collected from American crows at the same time points and added to 0.9 mL of serum-free DMEM. Coagulated blood from carrion crows was centrifuged at 1,300 × *g* for 5 min in MiniCollect vials (Greiner Bio-One, Alphen aan den Rijn, the Netherlands) to separate serum, and coagulated blood from American crows was centrifuged at 3,700 × *g* for 10 min to pellet clotted cells. Serum samples were stored at −80°C until further use.

Crows were examined for clinical signs twice daily for 14 dpi and euthanized under isoflurane anesthesia upon display of clinical signs. In addition, 2 birds per group of the carrion crows were euthanized at 4 dpi.

Necropsies were performed on all euthanized carrion crows; heart, liver, spleen, kidney, bone marrow, and brain samples were collected. A small section of each tissue was collected, weighed, and homogenized by using a metal bead in 1 mL of DMEM containing 100 U penicillin and 100 μg/mL streptomycin. The remaining portion of the tissues was collected in formalin for use in immunohistochemical staining.

### Determination of Virus Loads

We used quantitative real-time reverse transcription PCR (qRT-PCR) to determine virus titers in serum and tissue samples and TCID_50_ titration to calculate infectious virus titers in serum only. In brief, RNA was isolated from 50 μL of serum or 100 μL of homogenized tissue by using the MagNA Pure LC Total Nucleic Acid Isolation Kit (Roche, Almere, the Netherlands) and a MagNA Pure LC automated nucleic acid robotic workstation (Roche) according to the manufacturer’s instructions, and subsequently stored at −80°C. RNA copy numbers were quantified by using unmodified primers as previously described (*26*). The limit of detection of the assay was 9 (1.0 log_10_) RNA copies.

After log_10_ titration of serum samples on Vero E6 cells, cytopathic effect was determined at 5 dpi and TCID_50_ infectious titers were calculated by using the Spearman–Kärber method (*27*,*28*). An initial 1:10 dilution of serum resulted in a limit of detection of 10^1.8^ TCID_50_/mL.

### Immunohistochemistry

Paraffin sections (4-µm thick) of sagittal organ were processed for streptavidin–biotin–peroxidase immunohistochemical detection of nonstructural protein (NS) 3 antigen. Sections were deparaffinized in xylene, rehydrated in descending concentrations of ethanol, and incubated for 10 min in 3% H_2_O_2_ diluted in PBS to block endogenous peroxidase activity. Antigen exposure was performed by incubation at 121°C for 15 min in citrate buffer (10 mmol/L, pH 6.0). Sections were subsequently incubated overnight at 4°C with polyclonal goat anti-WNV NS3 protease (1:100; R&D Systems, Abingdon, UK) or isotype control (goat serum, 1:100; Dako, Eindhoven, the Netherlands) and then detected with polyclonal rabbit anti-goat IgG/HRP (Dako) antibody. Sections were counterstained with Mayer hematoxylin, mounted with Kaiser glycerin-gelatin, and analyzed by using a light microscope.

### Statistical Analyses

Survival curves were analyzed by using the log-rank (Mantel-Cox) test. Statistical analyses between >2 groups were performed by using Kruskal-Wallis 1-way analysis of variance; any significant differences were more closely analyzed between the groups by using the Mann-Whitney U test. A Bonferroni correction was applied to each p value, according to the number of comparisons (corrected p value of 0.05/10 = 0.005 for carrion crow peak viremia and organs of carrion crows euthanized on day 4; corrected p value of 0.05/6 = 0.008 for American crow peak viremia and organs of carrion crows euthanized due to illness). For all comparisons, each group had 6 crows, except for American crow groups that received FIN or Ita09 (n = 5).

## Results

### Illness and Death

**Figure 1:**
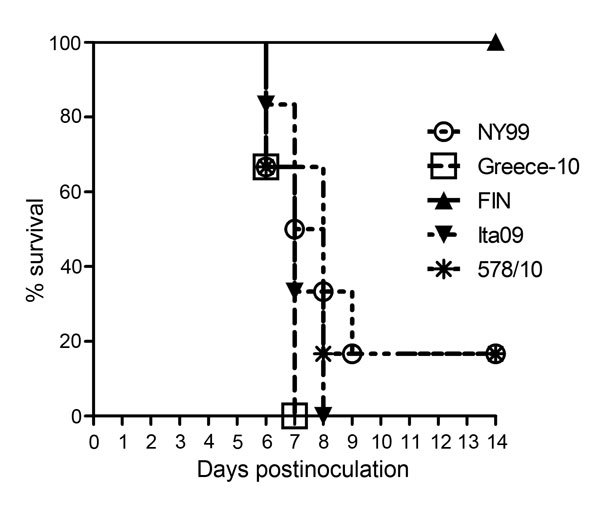
Survival rate for West Nile virus (WNV)–infected carrion crows after inoculation with 2,000 50% tissue culture infectious doses of WNV; each group (n = 6) was inoculated with a different strain. Crows were monitored daily for signs of disease through postinoculation day 14.

Signs of illness (e.g., lethargy, unresponsiveness, anorexia, and ruffled feathers) were observed among most crows within 9 dpi. All 6 carrion crows inoculated with Greece-10 or Ita09 died, and 5 (83%) of the 6 inoculated with NY99 or 578/10 died. All 6 carrion crows inoculated with strain FIN survived (Table 2). Survival curves of the infected birds showed a significant difference in survival between carrion crows infected with Ita09, Greece-10, NY99, or 578/10 and those infected with FIN (p = 0.005) (Figure 1). The median day of death was 7 dpi for carrion crows that died from infection with NY99, Greece-10, or Ita09 and 8 dpi for birds that died from infection with 578/10. All American crows inoculated with NY99 (n = 6) or Ita09 (n = 5) died, and all 5 crows inoculated with FIN survived (Table 3).

**Table 2:** Clinical profile for carrion crows experimentally infected with various West Nile virus strains*

Virus group	No. died/no. total (%)	Day of death, median dpi	Median peak viremia, viral RNA/mL serum (range); no. birds	Mean duration, d, of viremia ± SD; no. birds†	Mean day of peak viremia ± SD; no. birds†	Median peak viremia TCID_50_/mL (range); no. birds‡
NY99-4132	5/6 (83)	7	10^8.7^ (10^1.0^–10^10.0^); 6	5.2 ± 1.0; 5	4.3 ± 0.7; 5	10^7.4^ (10^1.8^–10^8.8^); 6
Nea Santa-Greece-2010	6/6 (100)	7	10^10.3^ (10^9.8^–10^11.7^); 6	5.7 ± 0.7; 6	4.5 ± 0.9; 6	10^7.8^ (10^7.3^–10^9.8^); 6
Italy/2009/FIN	0/6 (0)	NA	10^2.7^ (10^1.0^–10^5.9^); 6	2.7 ± 0.9; 3	6.7 ± 0.9; 3	10^1.8^ (10^1.8^–10^2.5^); 6
Ita09	6/6 (100)	7	10^9.7^ (10^8.0^–10^10.0^); 6	6.0 ± 1.2; 6	4.3 ± 0.7; 6	10^7.6^ (10^6.3^–10^8.8^); 6
578/10	5/6 (83)	8	10^8.4^ (10^6.0^–10^10.1^); 6	5.7 ± 1.8; 6	3.5 ± 0.9; 6	10^5.1^ (10^2.8^–10^8.5^); 6

*dpi, days postinoculation; NA, not applicable; TCID_50_, 50% tissue culture infectious dose. †Based on viral RNA titers. ‡Viral titers are expressed as log_10_ TCID_50_/mL of serum.

**Table 3:** Clinical profile of American crows experimentally infected with West Nile virus strains NY99-4132, Italy/2009/FIN, and Ita09

Virus group	No. died/no. total (%)	Median peak viremia, viral RNA/mL serum (range); no. birds	Median peak viremia, TCID_50_/mL serum (range); no. birds*
NY99-4132	6/6 (100)	10^9.6^ (10^9.1^–10^10.1^); 6	10^7.2^ (10^4.7^–10^7.2^); 6
Italy/2009/FIN	0/5 (0)	10^1.0^ (10^1.0^–10^6.9^); 5	10^1.8^ (10^1.8^–10^2.7^); 5
Ita09	5/5 (100)	10^8.8^ (10^8.0^–10^9.1^); 5	10^6.7^ (10^6.0^–10^7.5^); 5
*Virus titers are expressed as log_10_ 50% tissue culture infectious dose (TCID_50_)/mL of serum.			

### Viremia Profiles

**Figure 2:**
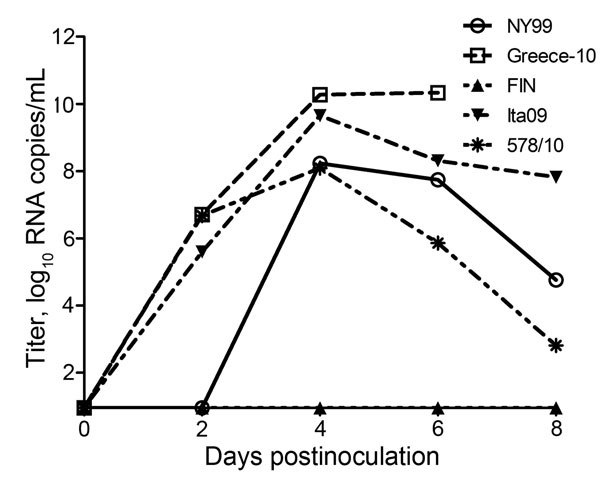
Viral RNA copy numbers for West Nile virus (WNV)–infected carrion crows after inoculation with 2,000 50% tissue culture infectious doses of WNV; each group (n = 6) was inoculated with a different strain. RNA copy numbers are represented as log-transformed medians. The assay had a detection limit of 9 (1.0 log_10_) RNA copies/mL of serum.

**Figure 3:**
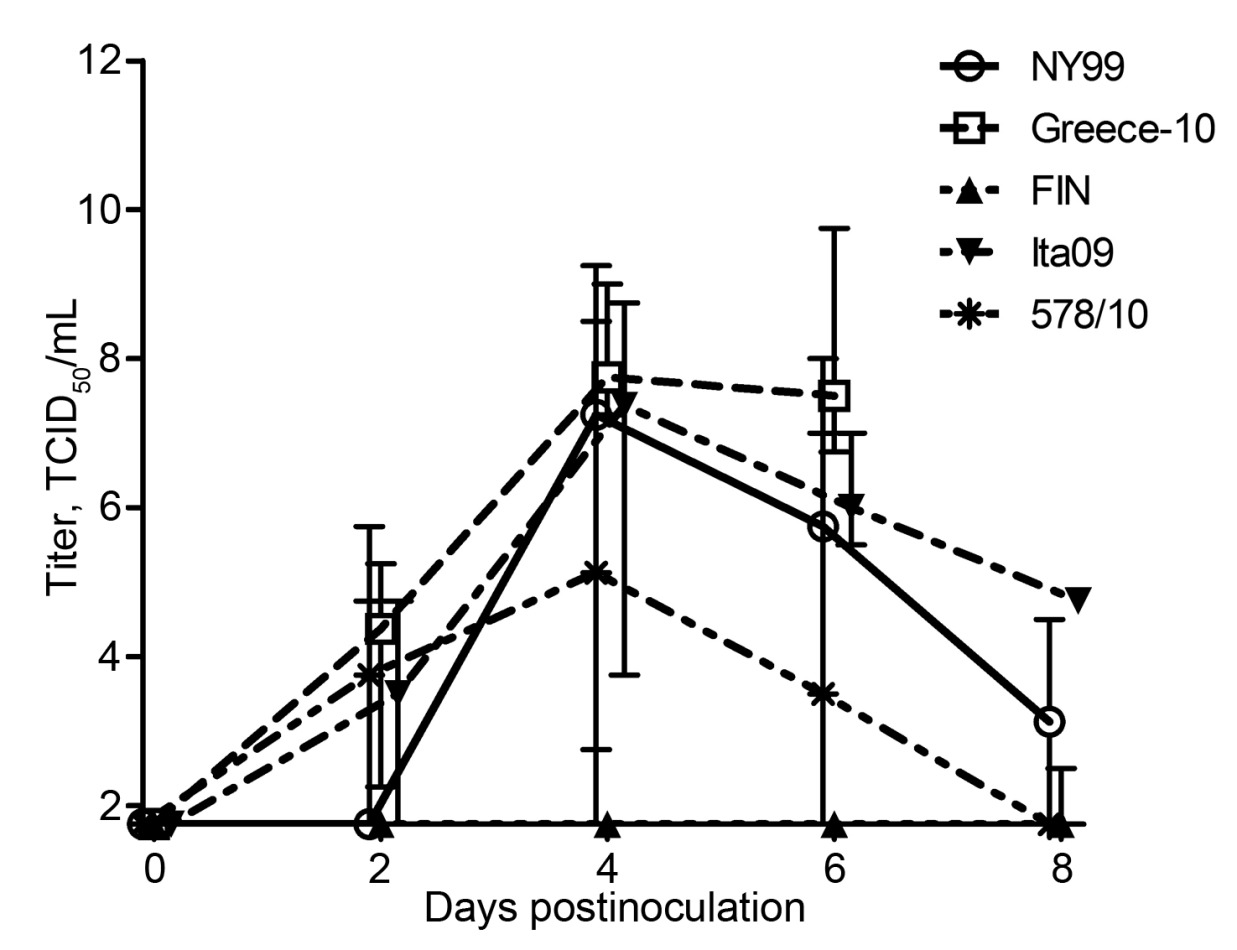
Infectious virus titer profiles for West Nile virus (WNV)–infected carrion crows after inoculation with 2,000 50% tissue culture infectious doses (TCID_50_) of WNV; each group (n = 6) was inoculated with a different strain. Infectious virus titers were determined by TCID_50_ titration and are represented as log-transformed medians; error bars indicate range. The assay had a detection limit of 1.8 TCID_50_/mL.

WNV viremia profiles were determined in terms of viral RNA (Table 2; Figure 2) and infectious virus titers in serum (Table 2; Figure 3) of infected carrion crows. In strain NY99–infected birds, the median peak viral RNA titer was 10^8.7^ RNA copies/mL of serum (range 10^1^–10^10.0^ [nontransformed values]), and the median peak infectious virus titer was 10^7.4^ TCID_50_/mL of serum (range 10^1.8^–10^8.8^); these values include 1 bird in which detectable viremia did not develop during the entire course of infection. The median peak viremia titer for Greece-10–infected birds was 10^10.3^ RNA copies/mL of serum (range 10^9.8^–10^11.7^) and 10^7.8^ TCID_50_/mL of serum (range 10^7.3^–10^9.8^). FIN-infected birds had median peak viremia titers of 10^2.7^ RNA copies/mL of serum (range 10^1^–10^5.9^) and 10^1.8^ TCID_50_/mL of serum (range 10^1.8^–10^2.5^); however, viremia was detectable in only 3 of 6 birds, and infectious virus could be isolated from only 1 bird. The median peak viremia titers for Ita09-infected birds were 10^9.7^ RNA copies/mL of serum (range 10^8.0^–10^10.0^) and 10^7.6^ TCID_50_/mL of serum (range 10^6.3^–10^8.8^). Birds infected with strain 578/10 had median peak viremia titers of 10^8.4^ RNA copies/mL of serum (range 10^6.0^–10^10.1^) and 10^5.1^ TCID_50_/mL of serum (range 10^2.8^–10^8.5^).

Strain Greece-10–infected birds had median peak viral RNA titers significantly higher than those for NY99-infected (p = 0.004), FIN-infected (p = 0.005), and 578/10-infected (p = 0.004) birds. Greece-10–infected birds also had median infectious virus titers significantly higher than those for FIN-infected birds (p = 0.003), but FIN-infected birds had RNA and infectious titers lower than those for Greece-10–infected (p = 0.005 and 0.003, respectively), Ita09-infected (p = 0.005 and 0.002, respectively), and 578/10-infected (p = 0.005 and 0.002, respectively) crows.

Viremia profiles were also determined for American crows infected with 3 of the 5 different WNV strains (Table 3). NY99-infected birds had median peak viremia titers of 10^9.6^ RNA copies/mL of serum (range 10^9.1^–10^10.1^) and 10^7.2^ TCID_50_/mL of serum (range 10^4.7^–10^7.2^). Detectable viremia developed in only 2 of the 5 FIN-infected birds, resulting in median peak viremia titers of 10^1.0^ RNA copies/mL of serum (range 10^1^–10^6.9^) and 10^1.8^ TCID_50_/mL of serum (range 10^1.8^–10^2.7^). Median peak viremia titers for Ita09-infected birds were 10^8.8^ RNA copies/mL of serum (range 10^8.0^–10^9.1^) and 10^6.7^ TCID_50_/mL of serum (range 10^6.0^–10^7.5^). American crows infected with strain NY99 had the highest median peak viral RNA and infectious virus titers, and FIN-infected birds had the lowest median titers (significant only when compared with each other: p = 0.008 and 0.006, respectively).

### Tissue Tropism

**Figure 4:**
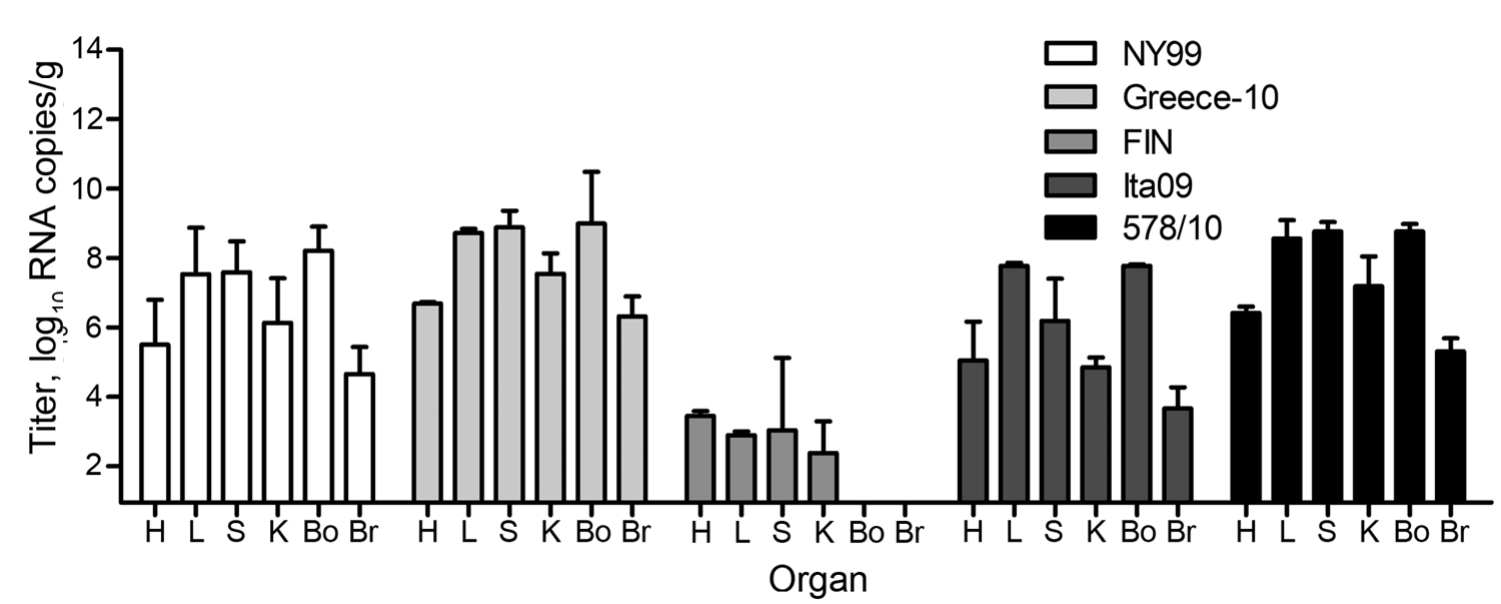
Viral RNA copy numbers in organs from 10 carrion crows (2 per group) euthanized 4 days after being experimentally infected with 1 of 5 different West Nile virus strains (n = 6, per group). Virus titers are represented as log-transformed medians; error bars indicate range. The assay had a detection limit of 9 (1.0 log_10_) RNA copies/g of tissue. H, heart; L, liver; S, spleen; K, kidney; Bo, bone marrow; Br, brain.

Virus loads were determined in the heart, liver, spleen, kidney, bone marrow, and brain of all birds. To assess the spread of virus to the different organs at the approximate peak of viremia, we euthanized 2 birds per group at 4 dpi. Virus was detected in all organs from these birds. On average, the highest viral RNA titers were detected in the liver, followed by the bone marrow, spleen, kidney, and heart; the lowest titers were found in the brain (Figure 4). Between the different virus strains, viral RNA titers were the highest in the organs of birds infected with strain Greece-10 or 578/10, followed by NY99 and Ita09; titers were significantly higher than those for birds infected with strain FIN (p = 0.005 for all). Virus distribution in FIN-infected birds was not consistent; viral RNA was undetectable in the bone marrow and brain of both birds tested on 4 dpi, and for 1 of these birds, viral RNA was also undetectable in the spleen.

**Figure 5:**
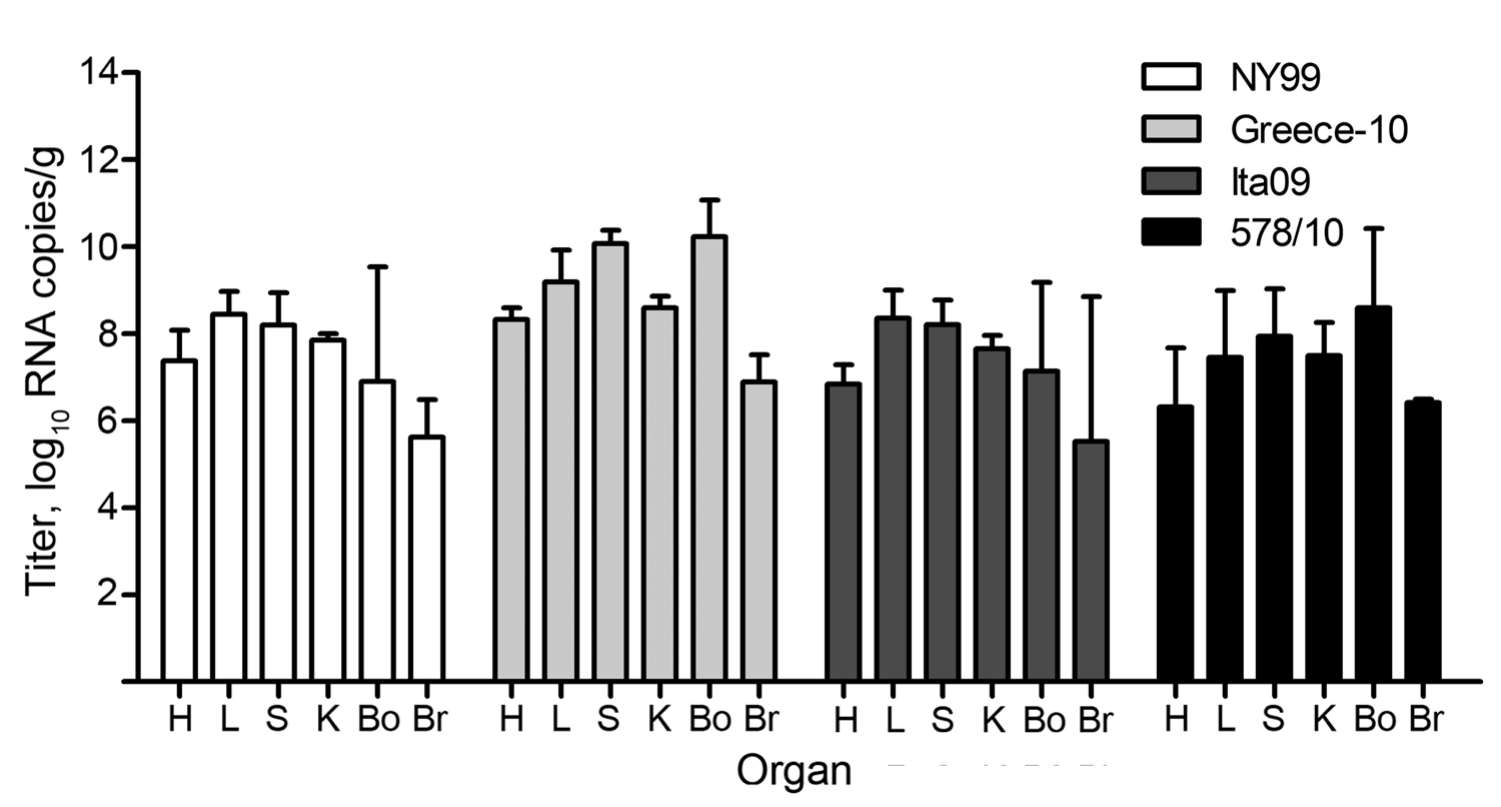
Viral RNA copy numbers in organs from 22 carrion crows euthanized because of illness after being experimentally infected with 1 of 4 different West Nile virus strains (n = 6, per group). Copy numbers are represented as log-transformed medians; error bars indicate range. The assay had a detection limit of 9 (1.0 log_10_) RNA copies/g of tissue. H, heart; L, liver; S, spleen; K, kidney; Bo, bone marrow; Br, brain.

Birds euthanized because of illness had virus present in all organs; in most cases, the spleen, liver, and bone marrow contained the highest average viral RNA load, followed by kidney and heart; the lowest average viral RNA titers were in the brain. Viral RNA titers in organs of Greece-10–infected birds were higher than those in organs of birds infected with the other viruses, but this observation was not statistically significant (Figure 5).

The 1 NY99-infected and 3 FIN-infected survivor birds that were free of viremia throughout the 8 days of blood sampling underwent necropsy at 14 dpi. Of interest, virus was present in all organs of the NY99-infected bird (median virus load of 10^3.1^ RNA copies/g of tissue) and in at least 3 of the 6 organs from FIN-infected birds (median virus load of 10^2.0^ RNA copies/g of tissue), showing that these birds did undergo productive WNV infection.

### Immunohistochemistry

Sections of organs from 2 birds necropsied at 4 dpi were stained with polyclonal anti-WNV NS3 to confirm replication of virus in the tissues and to exclude positive qRT-PCR detection due to spillover from blood at the approximate peak of viremia. Tissues most consistently positive for WNV antigen were the liver (80%), kidney (80%), bone marrow (80%), and spleen (78%); tissues least consistently positive for WNV antigen were heart (50%) and brain (10%) (Table 4). However, in terms of virus load, antigen was most abundant in the liver, bone marrow, and spleen. Overall, at 4 dpi, organs of birds most positive and most abundant for viral antigen were those infected by strains 578/10 and Greece-10, followed by NY99 and Ita09. The organs of FIN-infected birds were all negative for virus antigen at this time point.

**Table 4:** Immunohistochemical analysis of West Nile virus antigen distribution in experimentally infected carrion crows euthanized at 4 dpi*

Virus strain, bird no.	Heart	Liver	Spleen	Kidney	Bone marrow	Brain	Total score per bird	Average score per virus strain	No. positive organs/total no. organs
NY99-4132								9.0	
1	−	++	++	+	++	−	11.0		4/6
7	−	+	+	+/−	+	−	7.0		4/6
Nea Santa-Greece-2010								12.5	
1	+/−	++	++	+	++	+/−	13.0		6/6
7	+/−	++	++	+	++	−	12.0		5/6
Italy/2009/FIN								0	
1	−	−	−	−	−	−	0		0/6
7	−	−	−	−	−	−	0		0/6
Ita09								8.0	
1	+/−	++	++	+/−	+	−	10.0		5/6
7	−	++	ND	+/−	+	−	6.0		3/5
578/10								12.5	
1	++	++	++	+/−	++	−	13.0		5/6
7	+/−	++	++	+	++	−	12.0		5/6
Score per organ	7.0	23.0	20.0	12.0	21.0	1.0			
No. positive birds/total no. birds	5/10	8/10	7/9	8/10	8/10	1/10			
*Subjective determinations of the amount of antigen in each organ were made: negative (−), minimal (+/−), moderate (+), or abundant (++). Each determination was given a score from 0 to 3: negative (0), minimal (1), moderate (2), and abundant (3). ND = not determined. dpi, days postinoculation; ND, not determined.									

## Discussion

In this study, we assessed the susceptibility of carrion crows to different strains of WNV. First we demonstrated that carrion crows are susceptible to WNV infection by using the North American strain NY99, which has previously been shown to be highly virulent in American crows (*19*–*23*). In agreement with the findings in those studies, our results showed that infection of carrion crows with NY99 resulted in high viremia titers and death. In addition, virus had disseminated to the organs of infected birds by 4 dpi, further demonstrating the susceptibility of carrion crows to WNV infection, which appears to be very similar to that of American crows.

Next we studied the susceptibility of carrion crows to selected strains of WNV from Europe. We found that carrion crows are highly susceptible to infection with both lineage 1 and 2 WNV strains from Europe. In addition, we showed that susceptibility is strain-dependent. Of the 5 WNV strains tested, 4 led to death for 83%–100% of infected birds and to high viremia titers and abundant antigen in the organs of euthanized birds; however, birds inoculated with FIN did not die from infection, and, they had relatively low virus titers in the blood and no viral antigen in the organs at 4 dpi. A previous study describing the inoculation of carrion crows with WNV strains from France (Fr2000) and Israel (Is98) also suggested that carrion crows are susceptible to infection with WNV in a strain-dependent manner (*29*). The study showed death rates of 33% (Fr2000) and 100% (Is98) from the 2 strains, and viral RNA loads in serum, oral swab samples, and feathers of Is98-infected birds were higher than those of Fr2000-infected birds (*29*). Thus, WNV strains FIN and Fr2000 show a similar attenuation in carrion crows.

To more accurately assess the virulence of WNV strains from Europe, we inoculated American crows, a bird species known to be highly susceptible to WNV, with 2 of the 4 strains from Europe (Ita09 and FIN) and with strain NY99 from North America. Similar to what was seen with carrion crows, American crows infected with Ita09 had high peak viremia titers, and all succumbed to the infection, whereas those infected with FIN had low viremia titers, and all survived infection. Furthermore, it was demonstrated that the Greece-10 strain used in this study was also 100% lethal in American crows (A.C. Brault et al., unpub. data). In fact, American crows infected with Greece-10 (vs. the other strains used in this study) had the highest median peak viremia titers in terms of RNA and infectious virus (data not shown). These results show that in American crows, WNV strains (apart from FIN) from Europe are as virulent as the prototypic NY99 strain from North America.

The fact that susceptibility of birds to WNV can be strain-dependent was clearly demonstrated by the attenuated virulence phenotype of WNV strain FIN in carrion and American crows (this study) and in European jackdaws (*30*); FIN-infected crows consistently exhibited an absence of death, lower peak viremia titers, and less dissemination of virus to the organs at the approximate peak of viremia. A previous study showed that the introduction of a P249T amino acid substitution in the NS3 helicase of North American strain NY99 led to a highly attenuated phenotype, whereas a T249P substitution introduced in a low-virulence WNV strain resulted in a phenotype highly virulent to American crows (*22*). Four virus strains used in this study contain a proline at NS3-249, whereas FIN contains a threonine at this position (*31*). It is therefore likely that the attenuated phenotype of FIN is a result of this threonine amino acid at NS3-249, a mutation that could be relevant for at least 3 different species of birds in the family *Corvidae*. Studies in North American and European corvids are ongoing in order to test the relevance of the T249P substitution and several other mutations when introduced into the genome of WNV-FIN.

We have shown that bird susceptibility to WNV can be strain-dependent. However, susceptibility is also clearly related to host factors. As a whole, jackdaws were less susceptible than the carrion crows to the same selection of otherwise highly virulent WNV strains, and they had lower death rates and virus loads in blood and organs (*30*). Species susceptibility has been shown to differ within various avian families (*7*), including birds in the family *Corvidae*, of which, for example, the fish crow (*Corvus ossifragus*) was less susceptible to lethal WNV infection (*23*). Although the reasons for this varied susceptibility are not well understood, potential contributing factors may include host traits, such as genetic composition, immune response, and physiologic mechanisms (*23*).

A measure of the potential for transmission of virus to feeding mosquitoes is the level of infectious virus titers produced during viremia. The median peak serum titer of infectious virus was highest in Greece-10–infected carrion crows and lowest in FIN-infected carrion crows. Studies have shown that WNV titers of >10^5^ PFU/mL were considered infectious for *Culex pipiens* (*32*) and *Cx. quinquefasciatus* (*33*) mosquitoes. Considering this cutoff of 10^5^ PFU/mL or of 10^5.2^ TCID_50_/mL, according to a conversion factor of 1 TCID_50_ to 0.7 PFU (*34*), infectious titers obtained for carrion crows infected with Greece-10, Ita09, or NY99 would be sufficient for efficient transmission of virus to feeding mosquitoes. Carrion crows infected with strain 578/10 had median peak viremia titers slightly below this threshold (10^5.1^ TCID_50_/mL; Table 2), suggesting that the carrion crow may not be an efficient amplifier for this WNV strain. However, a possible explanation for the apparent low viremia titers in 578/10-infected birds could be that blood sampling was conducted on alternate days, possibly missing higher peak viremia titers of infectious virus. For the American crows, median peak viremia titers for Ita09 (Table 3) were slightly lower than those for carrion crows (Table 2). However, serum samples from American crows underwent 2 repeated freeze–thaw cycles, which could have resulted in the detection of lower infectious virus titers. Nonetheless, these results show that WNV strains from Europe can produce viremia titers in American crows that could be sufficient for efficient transmission to feeding mosquitoes. Nevertheless, reservoir competence studies involving the feeding of European mosquitoes on viremic WNV-infected carrion crows are needed to determine whether the carrion crow could indeed be a potential reservoir host and contributor to the WNV transmission cycle. 

We have shown that carrion crows, a species of bird ubiquitously found across Europe, are highly susceptible to WNV strains currently circulating in Europe. These birds could therefore potentially be useful as part of dead bird surveillance in the early detection of WNV in Europe. Future studies assessing the susceptibility of the closely related hooded crow (*Corvus cornix*) to WNV may also prove to be insightful, as this is the more predominant corvid species in eastern and southeastern Europe, where WNV is more common. The susceptibility of European birds to WNV has been demonstrated in multiple studies (*9*,*10*,*12*,*13*,*29*,*30*,*35*–*38*), however, it is peculiar that the number of WNV-associated deaths among birds in Europe is not as extensive as that among birds in North America. Possible explanations may be a lower reporting of bird deaths in Europe as compared with that in the United States or that other ecologic factors, such as mosquito competence, abundance, distribution or behavior, exert a limiting effect on the transmission of WNV in Europe.

Technical Appendix Methods for detecting preexisting West Nile virus neutralizing antibodies in crows captured for study.
