# Supplementary material for: Susceptibility of Carrion Crows to Experimental Infection with Lineage 1 and 2 West Nile Viruses
Source: Emerg Infect Dis. 2015 Aug;21(8):1357–65. doi: 10.3201/eid2108.140714 (PMC4517732; doi:10.3201/eid2108.140714)
Supplement: Technical Appendix — Methods for detecting preexisting West Nile virus neutralizing antibodies in crows captured for study. [file 14-0714-Techapp-s1.pdf]

Article DOI: http://dx.doi.org/10.3201/eid2108.140714

Susceptibility of Carrion Crows to Experimental Infection with Lineage 1 and 2 West Nile Viruses

Technical Appendix

Detection of Preexisting West Nile Virus Antibodies

To confirm that Carrion crows had not previously been exposed to WNV, the birds were bled before experimental infection and serum was tested for neutralizing antibodies by using tissue culture infectious dose 50 ($TCID_{50}$) neutralization assays. Serum was heat-inactivated at 56°C for 30 min, serially diluted 2-fold and incubated with an equal volume of virus (strain NY99, originally isolated from a dead Chilean flamingo at the Bronx Zoo in New York, obtained from the Health Protection Agency, Porton Down, UK; P5 on Vero E6 cells; accession AF196835.2) to a final concentration of 100 $TCID_{50}$/0.1 mL. Samples were incubated at 37°C for 1 h and subsequently added to an 80% confluent monolayer of Vero E6 cells in CELLSTAR 96-well plates (Greiner Bio-One, Alphen aan den Rijn, The Netherlands). Plates were incubated at 37°C for 5 days. Samples were read, and a 100% reduction in cytopathic effect, as compared with the serum-negative control, was used for the determination of neutralization. Detection of any neutralizing activity to WNV in the serum of any bird precluded its use for experimental inoculation.
